# Supplementary material for: Giant bulk photovoltaic effect driven by the wall-to-wall charge shift in WS2 nanotubes
Source: Nat Commun. 2022 Jun 10;13:3237. doi: 10.1038/s41467-022-31018-8 (PMC9187746; doi:10.1038/s41467-022-31018-8)
Supplement: Supplementary file 1 — Supplementary information [file 41467_2022_31018_MOESM1_ESM.pdf]

Supplementary Information for
Giant bulk photovoltaic effect driven by the wall-to-wall charge shift in WS_2 nanotubes

Bumseop Kim[1], Noejung Park[1*], Jeongwoo Kim[2*]

[1]*Department of Physics, Ulsan National Institute of Science and Technology, Ulsan, 689-798 Korea*

[2]*Department of Physics, Incheon National University, Incheon, 406-772 Korea*

Supplementary Note 1 Electric polarization of the zigzag SWNTs.

The unit of three-dimensional (3D) polarization density is C/m^2. For one-dimensional (1D) TMD nanotubes, we use 1D polarization density (*e*/*n*) where n is the unit chiral index. For a comparison with 3D systems, we consider a close-pack triangular lattice of nanotube bundles. The conversion formula from 1D polarization density (*e*/*n*) to 3D polarization density (C/m^2) is given as,

$$\mathbf{P}\ (\mathrm{C/m^2}) = \mathbf{P}\ (e/n) \cdot \frac{1.602 \cdot 10^{-19}\ \mathrm{C}/e}{A\ (\text{Å}^2)} \cdot n \cdot 10^{20}\ \text{Å}^2/\mathrm{m}^2$$

where A is the area of the close-pack triangular lattice.

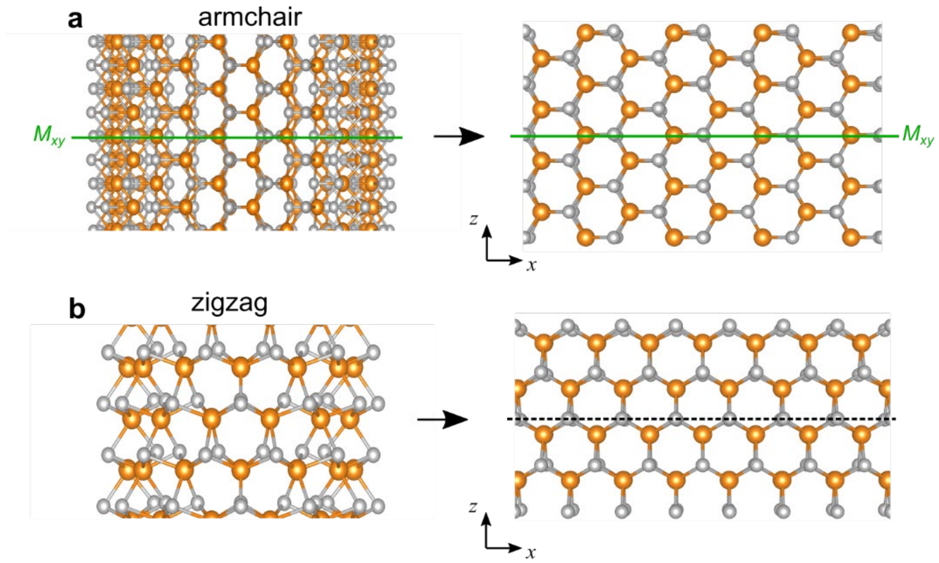

Supplementary Figure 1 Symmetry description of the zigzag and armchair SWNTs. When we unwind an armchair nanotube to a two-dimensional (2D) sheet as shown in Supplementary Figure 1a, the 2D sheet has a mirror symmetry (M_{xy}) denoted as green line. However, the 2D counterpart of a zigzag nanotube as illustrated in Supplementary Figure 1b does not the mirror symmetry.

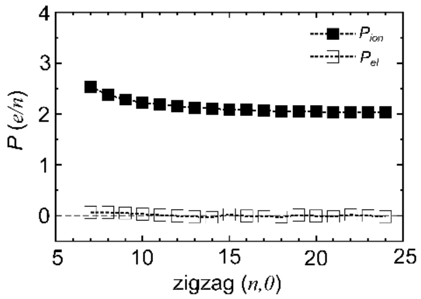

Supplementary Figure 2 Ionic and electronic polarization of the zigzag SWNTs. Calculated ionic polarization (P_{ion}) and electronic polarization (P_{el}) of the SWNT with various tube sizes.

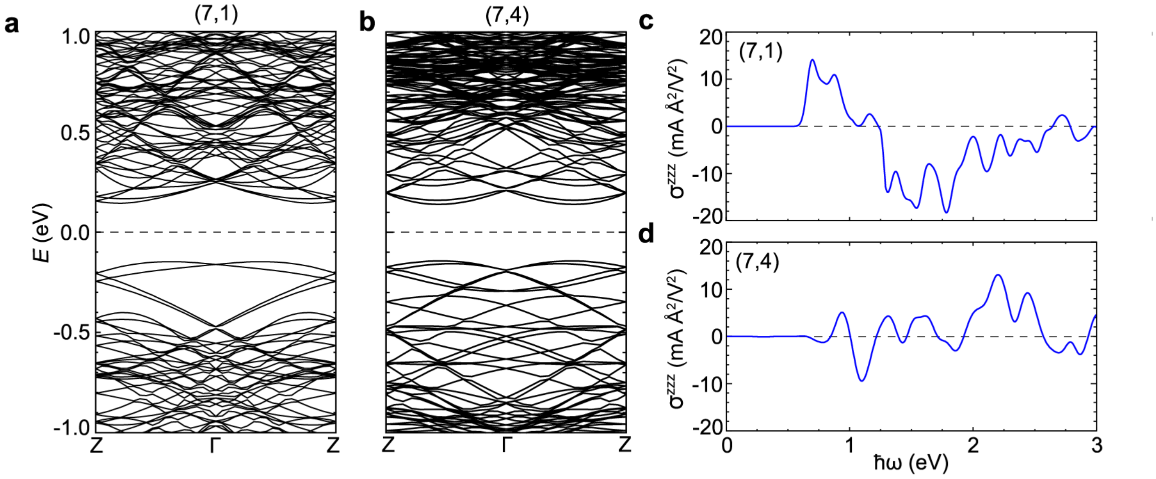

Supplementary Figure 3 Electronic structure and shift current spectra of chiral nanotubes. a–b Calculated band structure of (**a**) the (7,1) and (**b**) (7,4) SWNTs. **c–d** Calculated shift-current spectra of the (**c**) (7,1) and (**d**) (7,4) SWNTs with respect to the frequency of the applied light.

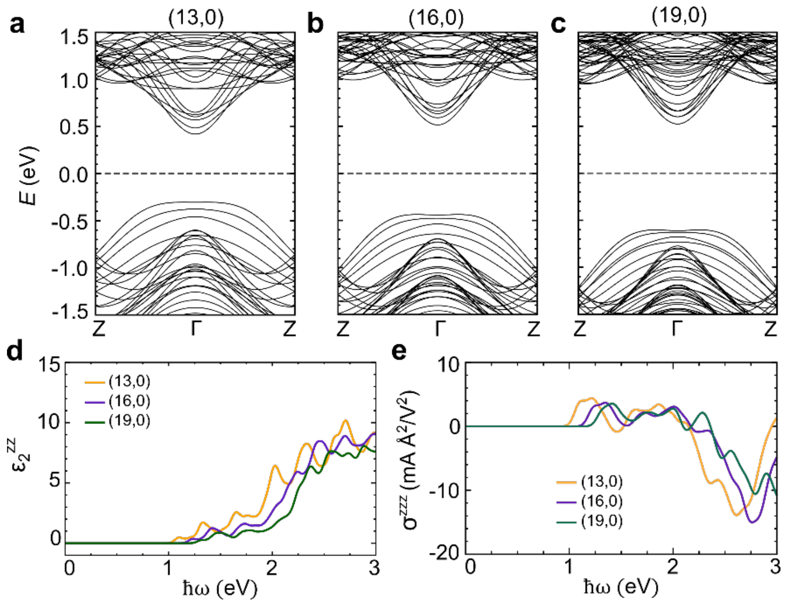

Supplementary Figure 4 Electronic structures of the zigzag SWNTs with various tube sizes. a–c Calculated band structures of the (**a**) (13,0), (**b**) (16,0), and (**c**) (19,0) SWNTs. **d** Imaginary parts of the dielectric constants of the (13,0), (16,0), and (19,0) SWNTs **e** Calculated shift current spectra of the (13,0), (16,0), and (19,0) SWNTs with respect to the frequency of the applied light.

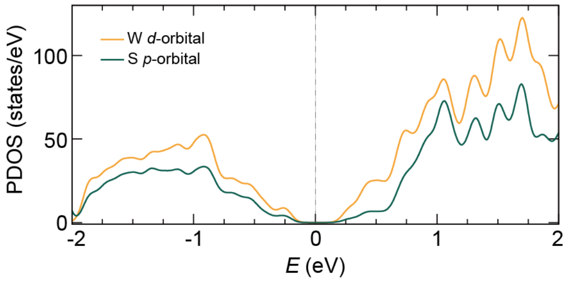

Supplementary Figure 5 Electronic structure of the zigzag SWNT. Orbital-projected density of states (PDOS) of the zigzag (10,0) SWNT.

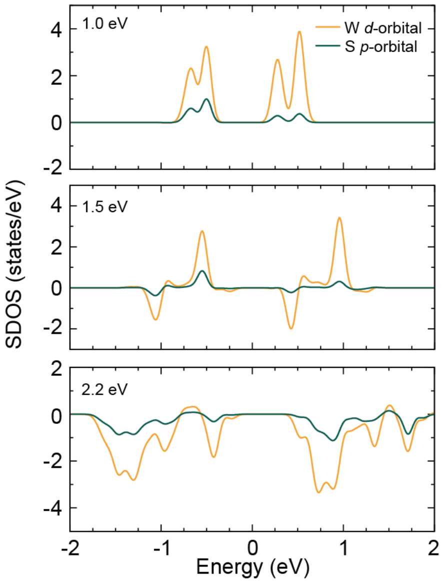

Supplementary Figure 6 Optoelectronic properties of the zigzag SWNT. Shift-current-weighted density of states of the zigzag (10,0) SWNT under various external fields ($\hbar\omega$ = 1.0, 1.5, 2.2 eV).

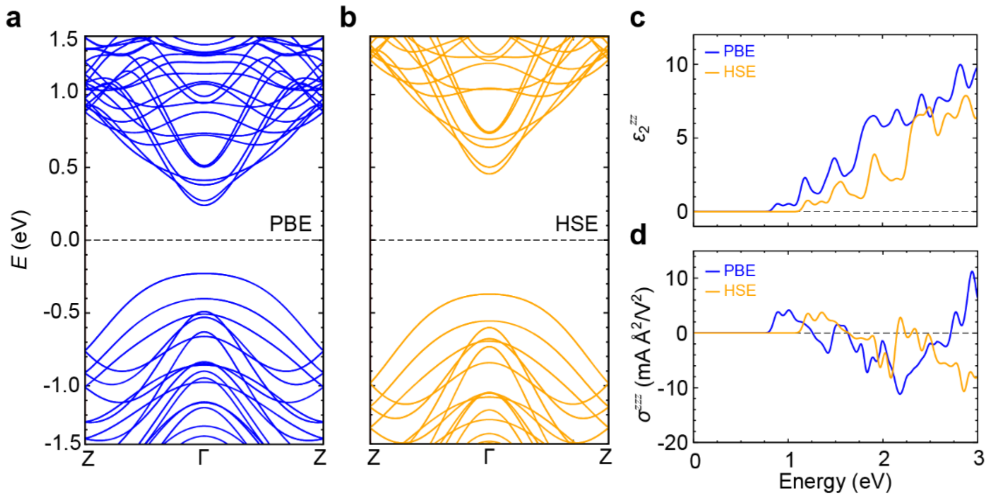

Supplementary Figure 7 Electronic structure and photovoltaic effect of the zigzag (10,0) SWNT using the hybrid HSE functional. a–b Calculated band structure of the SWNT using (**a**) the PBE and (**b**) the hybrid HSE functionals. **c** Imaginary parts of the dielectric constants of the zigzag (10,0) SWNT using the PBE or the HSE functionals. **d** Calculated shift-current spectrum of the zigzag (10,0) SWNT with respect to the frequency of the applied light using the PBE or the HSE functionals.

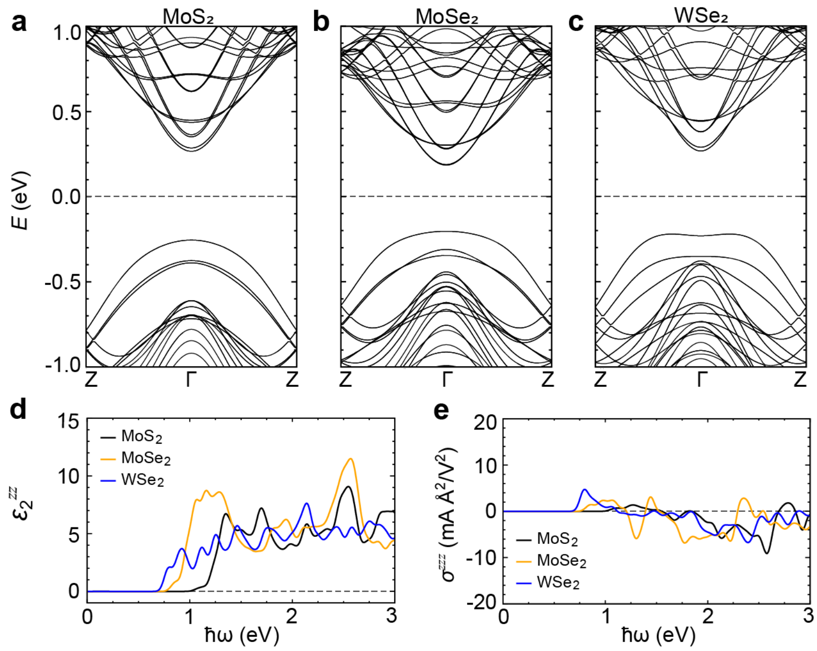

Supplementary Figure 8 Electronic and optoelectronic properties of various TMD SWNTs. a–c Calculated band structure of the zigzag (10,0) (**a**) MoS_2, (**b**) $MoSe_2$, and (**c**) WSe_2 SWNTs. **d** Imaginary parts of the dielectric constants of the zigzag (10,0) MoS_2, $MoSe_2$, and WSe_2 SWNTs. **e** Calculated shift current spectra of the zigzag (10,0) MoS_2, $MoSe_2$, and WSe_2 SWNTs with respect to the frequency of the applied light.

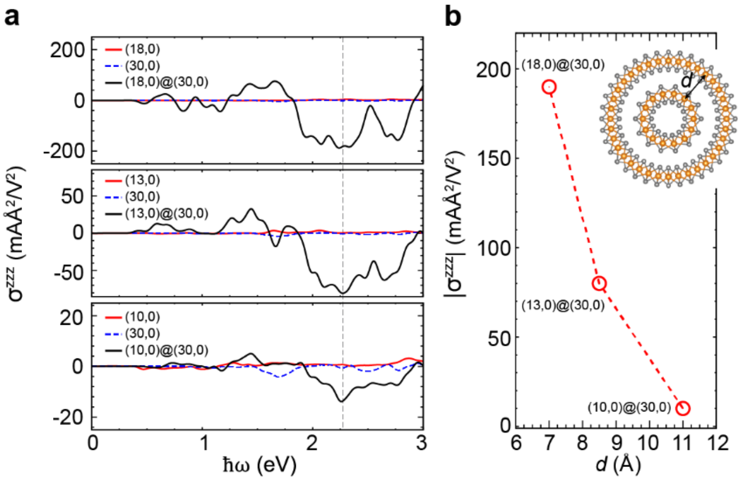

Supplementary Figure 9 The interwall effect depending the interlayer distance. A Calculated shift-current spectra of (10,0)@(30,0), (13,0)@(30,0), and (18,0)@(30,0) DWNTs with respect to the frequency of the applied light. **b** Maximum peak of shift-current spectra of (10,0)@(30,0), (13,0)@(30,0), and (18,0)@(30,0) DWNTs at 2.0 eV with respect to the distance between inner W and outer W atoms.

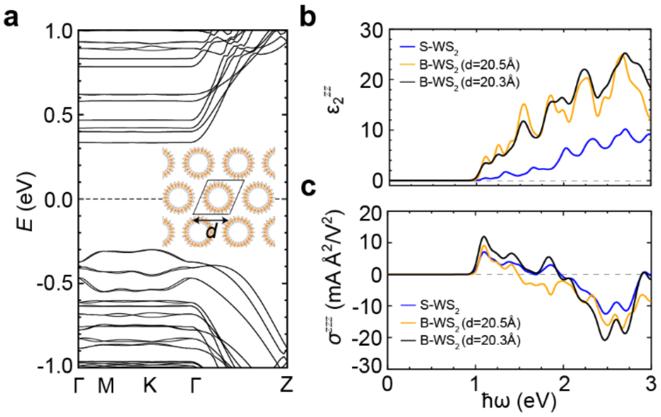

Supplementary Figure 10 Electronic and optoelectronic properties of a zigzag (13,0) SWNT bundle. a Calculated band structure of the SWNT bundle. The inset in Supplementary Figure 10a is the unit-cell geometry and the atomic structure of the SWNT bundle. **b** Imaginary parts of the dielectric constants of the SWNT (S-WS_2) the SWNT bundle (B-WS_2). **c** Calculated shift current spectra of the SWNT (S-WS_2) the SWNT bundle (B-WS_2) with respect to frequency of the applied light.

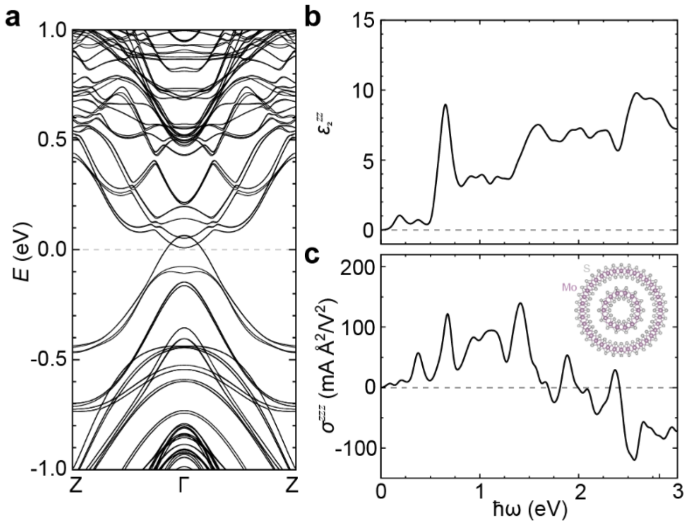

Supplementary Figure 11 Electronic and optoelectronic properties of MoS_2 (7,0)@(18,0) DWNT. **a** Calculated band structure of the MoS_2 DWNT. **b** Imaginary parts of the dielectric constants of the MoS_2 DWNT. **c** Calculated shift current spectra of the MoS_2 DWNT with respect to the frequency of the applied light. The inset indicates top view of the MoS_2 DWNT. The purple and gray spheres indicate the Mo (molybdenum) and S (sulfur) atoms, respectively.

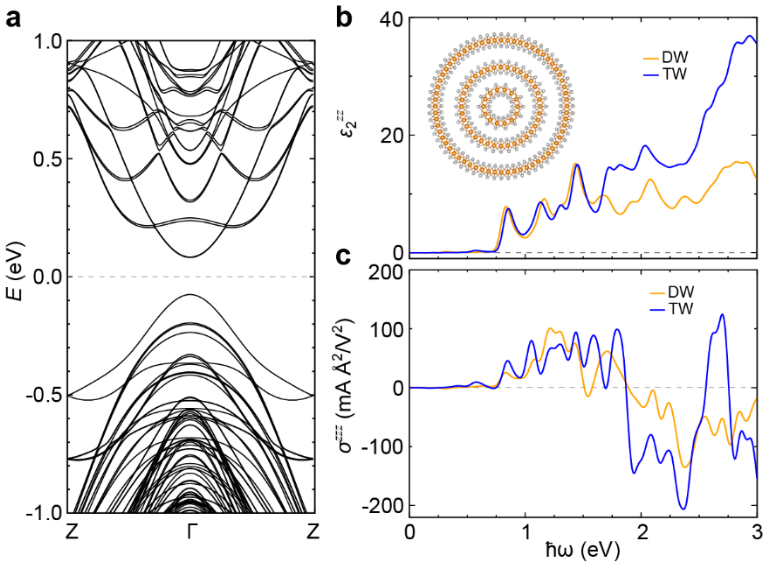

Supplementary Figure 12 Comparison of the optoelectronic properties of the DWNT and that of the triple-walled nanotube. a Calculated band structure of a (7,0)@(18,0)@(32,0) WS_2 triple-walled nanotube (TWNT) without spin-orbit coupling calculation. **b** Imaginary parts of the dielectric constants of the DWNT and the TWNT without spin-orbit coupling calculation. The inset is top view of the TWNT. **c** Calculated shift current spectra of the DWNT and the TWNT with respect to the frequency of the applied light without spin-orbit coupling.

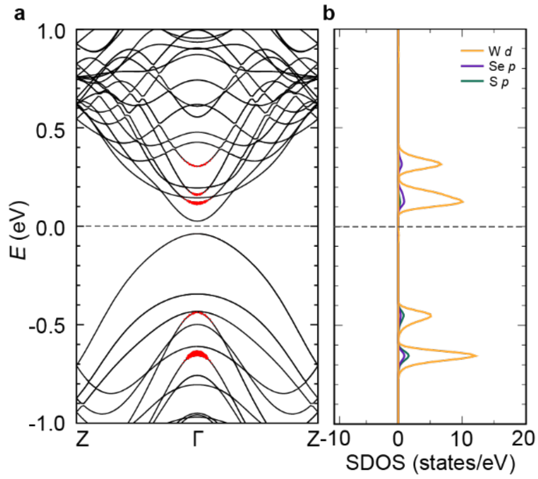

Supplementary Figure 13 Electronic structure and shift-current-weighted density of states of Janus-type WSSe SWNT. a Calculated band structures of the zigzag (10,0) WSSe SWNT. The peak indicated by the blue downward arrow in Fig. 4a corresponds to the transition between the valence and the conduction bands denoted by red dots. **b** Shift-current-weighted density of states (SDOS) of the WSSe SWNT corresponding to the transition between the red-dotted states of (**a**).

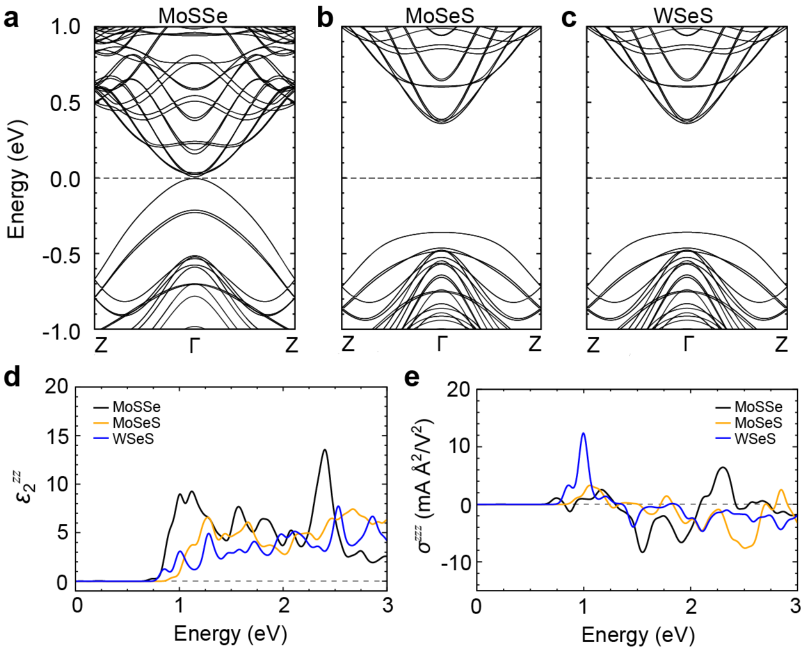

Supplementary Figure 14 Electronic and optoelectronic properties of various Janus-type SWNTs. a–c Calculated band structure of the zigzag (10,0) MoSSe, MoSeS and WSeS SWNTs. **d** Imaginary parts of the dielectric constants of the zigzag (10,0) MoSSe, MoSeS and WSeS SWNTs **e** Calculated shift current spectra of the zigzag (10,0) MoSSe, MoSeS and WSeS SWNTs with respect to the frequency of the applied light.

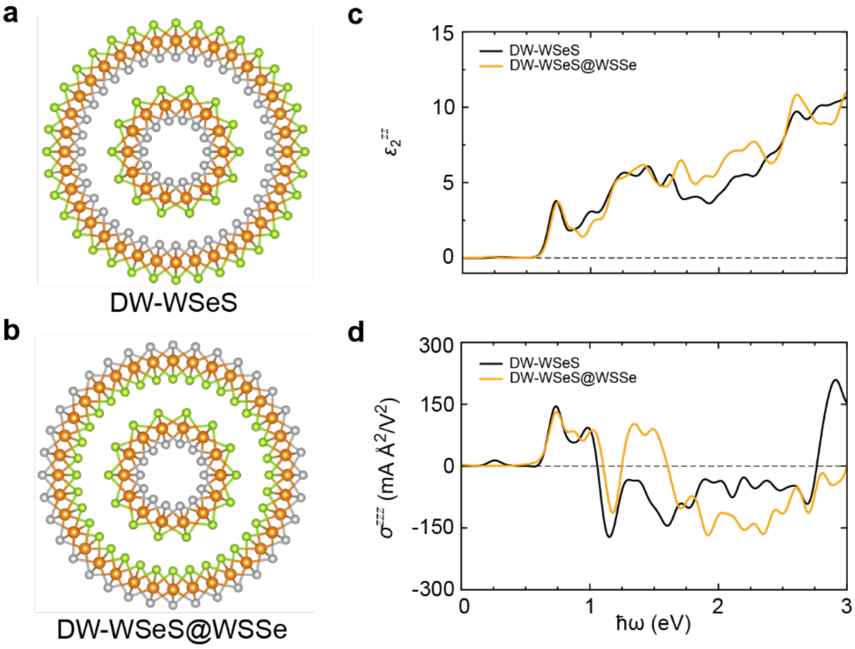

Supplementary Figure 15 Electronic and optoelectronic properties of various Janus DWNTs. a–b Top view of the (7,0)@(18,0) WSeS DWNT (DW-WSeS) and the (7,0)@(18,0) WSeS@WSSe DWNT (DW-WSeS@WSSe). **c** Imaginary parts of the dielectric constants of the DW-WSeS and DW-WSeS@WSSe. **d** Calculated shift current spectra of the DW-WSeS and DW-WSeS@WSSe with respect to the frequency of the applied light.

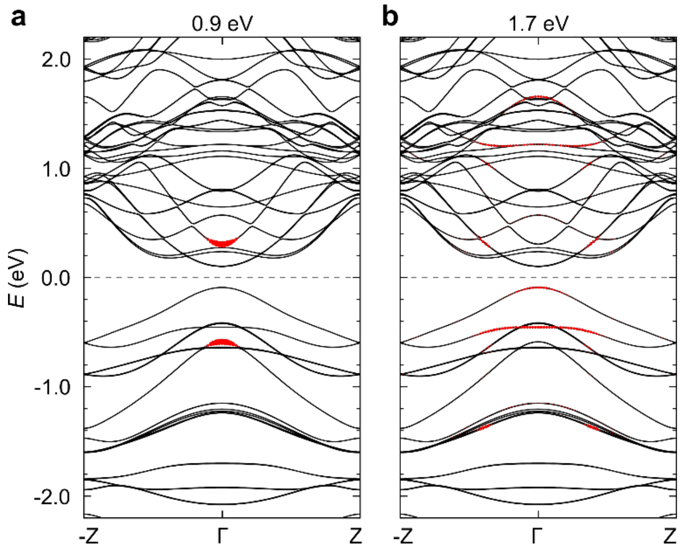

Supplementary Figure 16 n,k-resolved absorption of the zigzag (7,0) SWNT. a–b n,k-resolved absorption of the SWNT for external fields with (**a**) $\hbar\omega$ = 0.9 eV and (**b**) $\hbar\omega$ = 1.7 eV.

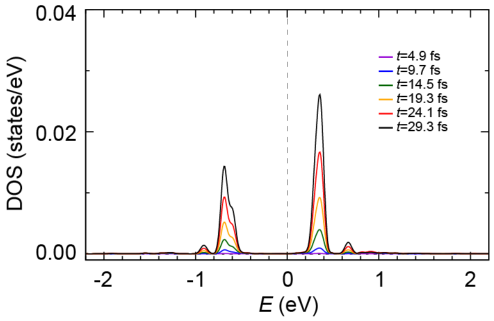

Supplementary Figure 17 Electronic structure of the excited states of the zigzag SWNT.

Time evolution of the electron/hole-carrier density of states of W atoms in the zigzag (7,0) SWNT without atomic-motion.

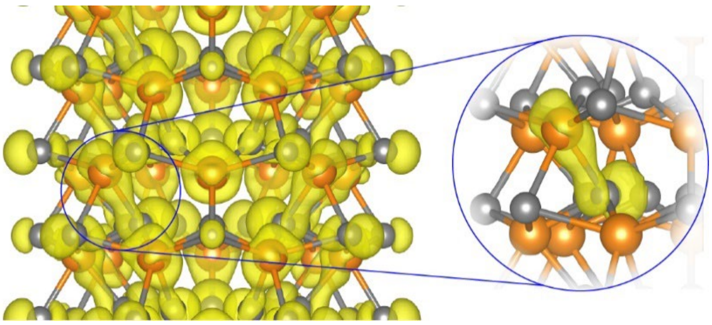

Supplementary Figure 18 Real space representation of the carriers of the zigzag SWNT.

Partial charge density of bonding state near -2 eV in the zigzag (7,0) SWNT.

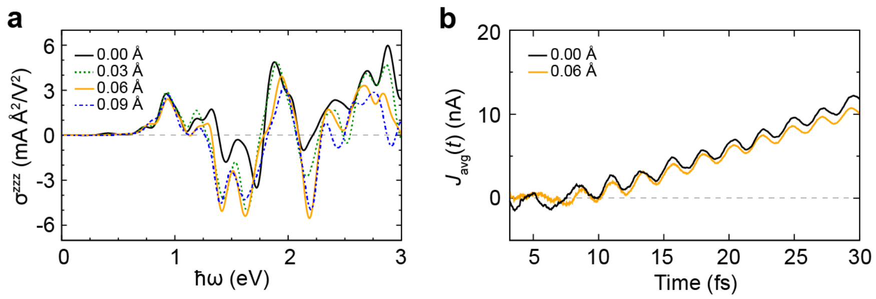

Supplementary Figure 19 The effect of displacement of the outer S atom on the shift-current spectra and the time-averaged second-order currents in the zigzag (7,0) SWNT. **a** Variation of the shift current induced by the displacement of the S atoms from their equilibrium positions in the SWNT. **b** Calculated time-averaged second-order currents of the SWNT under an external field with an intensity of 6.05×10^{10} W/cm^2 and frequency of $\hbar\omega = 0.9$ when the outer S atoms are moved from their equilibrium positions (0.06 Å).

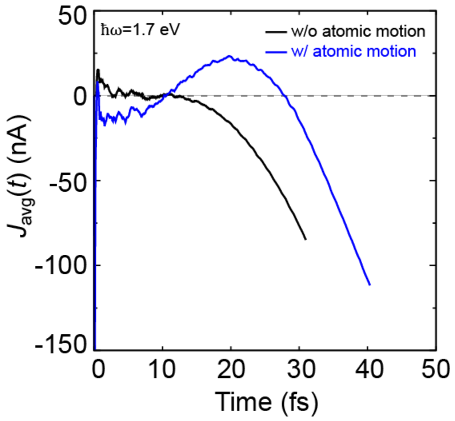

Supplementary Figure 20 Time-dependent density functional theory calculations with different light frequency. The time-averaged second-order currents in the zigzag (7,0) SWNT with the effect of atomic motion with respect to 1.7 eV frequency light.
